# Supplementary material for: Denitrification is a community trait with partial pathways dominating across microbial genomes and biomes
Source: Nat Commun. 2025 Oct 28;16:9495. doi: 10.1038/s41467-025-65319-5 (PMC12569097; doi:10.1038/s41467-025-65319-5)
Supplement: Supplementary file 2 — Description of Additional Supplementary Files [file 41467_2025_65319_MOESM2_ESM.pdf]

Description of Supplementary Data Files

File Name: Supplementary Data 1
Description: Total sum of denitrification genes in metagenomes derived from different biomes. For details, see the tab Supplement description.

File Name: Supplementary Data 2
Description: Database of assemblies used for surveying prevalence of denitrifier types. For details, see the tab Supplement description.

File Name: Supplementary Data 3
Description: Presence or absence of genes involved in different reduction or oxidation reactions in bacterial genomes. Column names for genes follow standard gene names.

File Name: Supplementary Data 4
Description: Sugar and acid preference inferred from genomes using KEGG orthologue ratios annotated using enrichM. For definitions, see the tab Supplement description.

File Name: Supplementary Data 5
Description: Pathway score completeness cutoffs used for denoting presence or absence of ability to use a given substrate in GapMind.

File Name: Supplementary Data 6
Description: Genomic density of transporters (TCDB) and transcription factors (deepTfactor) in bacterial genomes. For details, see the tab Supplement description.

File Name: Supplementary Data 7
Description: Abiotic environmental tolerances predicted from genomes using genomeSPOT. All values follow https://github.com/cultivarium/GenomeSPOT, except range, which was calculated as the difference between maximum and minimum.

File Name: Supplementary Data 8
Description: Results from gRodon analysis. Columns are defined as at https://microbialgamut.com/gRodon-vignette.

File Name: Supplementary Data 9
Description: Metagenomes and metadata used in GraftM-based global meta-analysis of denitrifier prevalence. For definitions, see the tab Supplement description.

File Name: Supplementary Data 10
Description: Metadata for metatranscriptomes, including transcript ratios. For definitions, see the tab Supplement description.

File Name: Supplementary Data 11
Description: Summary of counts and relative abundance of assemblies with different denitrification enzyme genes by phylum. For details, see the tab Supplement description.

File Name: Supplementary Data 12
Description: Counts of *nosZ* in metagenomes, separated by clade and biome. For details, see the tab Supplement description.
